# Assessing the Association between Serum Ferritin, Transferrin Saturation, and C-Reactive Protein in Northern Territory Indigenous Australian Patients with High Serum Ferritin on Maintenance Haemodialysis

**DOI:** 10.1155/2017/5490963

**Published:** 2017-01-24

**Authors:** Sandawana William Majoni, Paul D. Lawton, Federica Barzi, Alan Cass, Jaquelyne T. Hughes

**Affiliations:** ^1^Royal Darwin Hospital, Department of Nephrology, Division of Medicine, Tiwi, Darwin, NT, Australia; ^2^Northern Territory Medical Programme, Flinders University School of Medicine, Tiwi, Darwin, NT, Australia; ^3^Wellbeing and Preventable Chronic Disease Division, Menzies School of Health Research, Charles Darwin University, Casuarina, NT, Australia

## Abstract

*Objective*. To determine the significance of high serum ferritin observed in Indigenous Australian patients on maintenance haemodialysis in the Northern Territory, we assessed the relationship between ferritin and transferrin saturation (TSAT) as measures of iron status and ferritin and C-reactive protein (CRP) as markers of inflammation.* Methods*. We performed a retrospective cohort analysis of data from adult patients (≥18 years) on maintenance haemodialysis (>3 months) from 2004 to 2011.* Results*. There were 1568 patients. The mean age was 53.9 (11.9) years. 1244 (79.3%) were Indigenous. 44.2% (*n* = 693) were male. Indigenous patients were younger (mean age [52.3 (11.1) versus 57.4 (15.2), *p* < 0.001]) and had higher CRP [14.7 mg/l (7–35) versus 5.9 mg/l (1.9–17.5), *p* < 0.001], higher median serum ferritin [1069 *µ*g/l (668–1522) versus 794.9 *µ*g/l (558.5–1252.0), *p* < 0.001], but similar transferrin saturation [26% (19–37) versus 28% (20–38), *p* = 0.516]. We observed a small positive correlation between ferritin and TSAT (*r*^2^ = 0.11, *p* < 0.001), no correlation between ferritin and CRP (*r*^2^ = 0.001, *p* < 0.001), and positive association between high serum ferritin and TSAT (*p* < 0.001), Indigenous ethnicity (*p* < 0.001), urea reduction ratio (*p* = 0.001), and gender (*p* < 0.001) after adjustment in mixed regression analysis.* Conclusion*. Serum ferritin and TSAT may inadequately reflect iron status in this population. The high ferritin was poorly explained by inflammation.

## 1. Introduction

The effective treatment of anaemia in patients on maintenance haemodialysis (MHD) includes identification and correction of iron deficiency [1], use of erythropoiesis stimulating agents (ESA) as necessary, and achieving dialysis adequacy. Interpretation of iron status from most guidelines on anaemia management in people on MHD has been mainly based on transferrin saturation [the ratio of serum iron to the total iron binding capacity (TIBC) as a percentage, TSAT] and serum ferritin. Other measures of iron status have also been examined and include percentage hypochromic red cells (PHRC), reticulocyte haemoglobin content, and soluble transferrin receptor [2–4]. The accurate determination of iron status is critical in patients dependent on maintenance haemodialysis in order to avoid overtreatment resulting in iron overload and minimising continuing anaemia from undertreatment of iron deficiency.

The combination of serum ferritin levels and TSAT are commonly used worldwide in Renal Anaemia Management guidelines [2, 5]. Low TSAT and low ferritin are indicative of iron deficiency. However, recent published evidence suggests that TSAT is a better marker of iron status than ferritin and is more predictive of the response to treatment of anaemia in MHD patients [6]. Therapeutic supplementation of iron is provided to MHD patients until predetermined targets of these measures are achieved. Internationally, the target levels vary (Table 1). In our analysis, we used the ferritin and TSAT target levels in the Kidney Disease: Improving Global Outcomes (KDIGO) and Caring for Australasians with Renal Insufficiency (CARI) guidelines and their modifications to suit our local needs developed using the results from the DRIVE studies because these guidelines encompass international consensus and apply to our region, respectively.

We recently reported excessive serum ferritin concentrations among Indigenous Australian patients on MHD [7]. However, anaemia management guidelines worldwide presently do not guide iron supplementation in settings of high ferritin, including when high ferritin, an acute phase reactant, occurs in the setting of inflammation. The use of either measure alone has significant problems with lack of accuracy and has been discouraged in a recent special report from the United Kingdom National Institute for Health and Care Excellence (NICE) [8]. There is accumulating evidence that high serum ferritin concentrations are associated with high rates of hospitalisations, cardiovascular disease, and mortality. Given the concerns of high dose iron supplementation in ESKD among populations with high background serum ferritin levels and high comorbid cardiovascular disease, in this study, we examined the relationship between serum ferritin levels and TSAT as measures of iron status and serum ferritin levels and C-reactive protein (CRP) as markers of inflammation among Indigenous Australian patients dependent on MHD [7]. The analysis tested the hypothesis that (1) the high serum ferritin levels and TSAT may be inadequate as measures of iron status and (2) the high serum ferritin levels had no association with CRP as an inflammatory marker in Indigenous Australian patients from the Northern Territory who were on MHD.

## 2. Materials and Methods

### 2.1. Study Design and Participant Selection

We performed a retrospective cohort analysis of prospectively collected data for the 8-year period from 2004 to 2011 from the renal units serving a majority of Indigenous Australian population of Northern Australia. Adult patients over 18 years of age who had been on maintenance haemodialysis for at least 3 months were included in the analysis.

### 2.2. Data Source and Selection

Deidentified data were extracted from the Renal Anaemia Management (RAM) database which contained data collected prospectively every month from all renal units. The RAM database is a system developed by ©Syreon Corporation: Vancouver, BC, Canada, and was maintained by Janssen-Cilag© Pty Limited from all the renal units.

The data from the RAM database was imported into Stata for exploration for errors and consistence. Data quality checks for data integrity was performed that no significant information was lost during the transfer. Only variables relevant to the aims of the analysis were included in the final dataset.

### 2.3. Statistical Analysis

Exploratory analysis was performed for each variable summarising the data as means and their standard deviations (SD) for continuous normally distributed variables and medians and interquartile ranges (IR) for variables with skewed distributions. A summary analysis was also performed by ethnicity. Pearson's product-moment correlation was run to assess the relationship between (1) ferritin and TSAT as measures of iron status and (2) ferritin and CRP as markers of inflammation.

As part of building a statistical model to assess the relationship between ferritin and TSAT and CRP, a bivariate analysis of the relationship between ferritin and each of the variables was first performed. As the data was longitudinal with multiple observations for each variable and some of them unequally balanced, analyses were carried out using mixed models approach including a random intercept and a random effect to take account of intercorrelation between measurements taken over time for the same patient. Associations of ferritin with the variables were therefore assessed by (1) mixed regression analysis with ferritin as a continuous dependent variable and TSAT as the primary predictor variable and the rest of the variables as covariates and (2) mixed multinomial logistic regression analysis for categorical data with ferritin as a categorical variable as outlined below.

For fitting a regression model to assess the association between ferritin and TSAT with ferritin as a continuous dependent variable, a square root transformation of the ferritin levels was required as the distribution of ferritin was positively skewed. A variance component model was performed to assess if there was sufficient variance represented at a higher level in the data to justify the use of the mixed model approach. The currently accepted rule is that at least 10% of the total variance needs to be represented at a given level [9]. We used the level of the RAMID (level 2) and the level of the observations (level 1) to determine the total variance. Using only ferritin with no predictors, an empty model was created giving the variance component model estimates at level 2 (i.e., RAMID) and at level 1 (i.e., observations). Estimates from the variance components model were used to calculate the Intraclass Correlation Coefficient (ICC) which was determined as level 2 (RAMID) variance divided by level 2 (RAMID) variance + level 1 (observations) variance. The ICC was 444.3577^2^/(444.3577^2^ + 521.7812^2^) = 0.42037432. This means that approximately 42% of the total variance in the ferritin levels was represented at the level of the RAMID which is well above the 10% expected to justify using the mixed model. TSAT was then added to the model as the primary predictor followed by the other covariates to build a final multivariable model. Interaction between variables was also assessed, particularly the interaction between ethnicity and the other variables, using product terms to determine any potential interaction among the predictor variables.

For the second part of the assessment ferritin was categorized into 4 categories as low (less than 100 *µ*g/l), “within treated range” (100–800 *µ*g/l), high (800–1200 *µ*g/l), and very high (>1200 *µ*g/l) according to our local guidelines [7] to assess if there were any associations between the different categories of ferritin and TSAT, CRP, and the other variables. TSAT was also categorized as low (<20%), “within treated range” (≥20% to ≤30%), high (>30% to <50%), and very high (≥50%) and CRP as low (<10 mg/l) and high (≥10 mg/l).

We also performed complementary sensitivity analyses splitting the sample by ethnicity into Indigenous patients versus non-Indigenous patients in order to examine the association between ferritin and CRP and ferritin and infused iron dose in participants where the infused dose of iron was available.

All analyses were performed using Stata (R) version 13.1 (SE Copyright 1985–2013).

## 3. Results

There were 1568 adult patients in the study with a mean age of 53.9 (11.9) years; 79.3% (*n* = 1244) were Indigenous Australian patients; and 44.2% were male (*n* = 693). Indigenous patients were younger than non-Indigenous [52.3 (11.1) years versus 57.4 (15.2) years, *p* < 0.001]. The commonest comorbidities in Indigenous patients were hypertension (61.9%), diabetes mellitus (66.6%), and coronary artery disease (61.6%). 

Compared with non-Indigenous patients, Indigenous Australian patients had lower haemoglobin (*p* < 0.001), higher serum ferritin levels (*p* < 0.001), CRP (*p* < 0.001), URR (*p* = 0.010), and ESA weekly dose (*p* < 0.001). There were no differences for phosphate (*p* = 0.163), calcium and phosphate product (*p* = 0.173), BMI (*p* = 0.259), TSAT (*p* = 0.516), and KT/V (*p* = 0.171) (Table 2).

### 3.1. Correlation between Ferritin and TSAT, CRP, and Other Covariates

**Figure 1:**
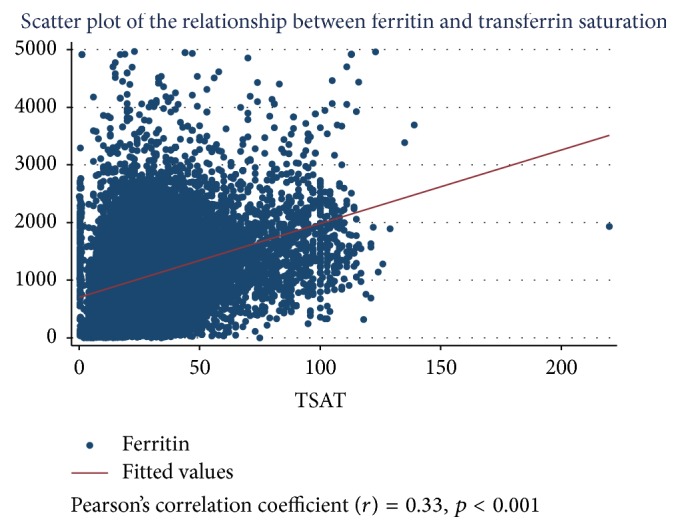
Scatter plot of the relationship between serum ferritin concentration and TSAT.

**Figure 2:**
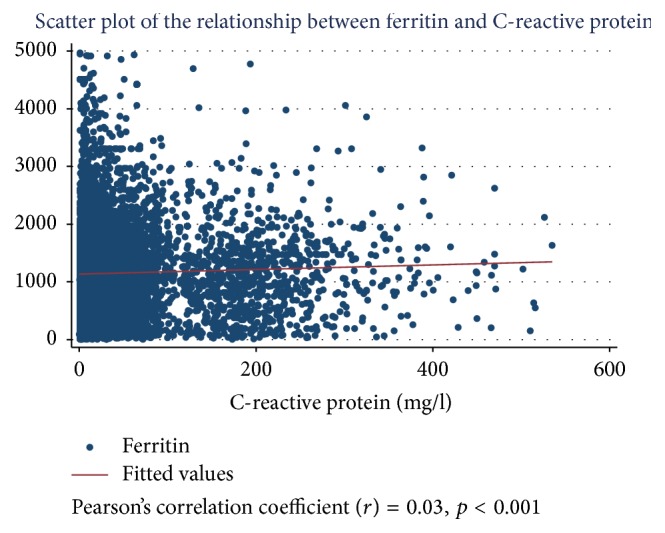
Scatter plot of the relationship between serum ferritin concentration and CRP.

Ferritin was positively correlated with TSAT (*r*^2^ = 0.11, *p* < 0.001) and had a significant association with URR (*p* < 0.001) and KT/V (*p* = 0.024). Ferritin was inversely associated with non-Indigenous ethnicity compared to Indigenous (*p* < 0.001), older age (*p* < 0.001), and higher BMI (*p* = 0.009). There was no correlation observed between ferritin and CRP (*r*^2^ = 0.001, *p* < 0.001) (Table 3 and Figures 1 and 2).

### 3.2. Mixed Regression Model of Serum Ferritin with TSAT as Predictor Variable

A final model developed by stepwise sequential selection and addition of variables with a significance level of *p* < 0.25 from the bivariate analysis included no interaction terms as there was no significant interaction between the different covariates. After adjusting for all the covariates, there remained a statistically significant association between ferritin and TSAT (*p* < 0.001), ethnicity (*p* < 0.001), and URR (*p* = 0.001) (Table 4). There was no significant association with CRP.

On mixed multinomial logistic regression, categories of ferritin were positively associated with “within treated range” and higher categories of TSAT (*p* < 0.001), Indigenous ethnicity (*p* < 0.001), and male gender (*p* < 0.001). There was no association between ferritin categories and all CRP categories (Table 5).

We found that although ferritin levels and CRP were higher in Indigenous Australian patients, the sensitivity analyses of the examination of the association between ferritin and CRP and ferritin and infused iron dose in participants where the infused dose of iron was available did not alter the findings observed in the combined cohort.

## 4. Discussion

Indigenous MHD patients in this study had lower haemoglobin, higher ferritin, CRP, and ESA weekly dose, despite similar concentration of TSAT and similar degree of measures of dialysis adequacy than non-Indigenous patients. Two key findings in this analysis were as follows: (1) the high ferritin levels in our patients are only partly explained by iron levels (TSAT) and (2) high ferritin levels in our patients are likely to be poorly explained by inflammation given the poor association between ferritin and CRP.

Recent evidence has shown that TSAT (compared with serum ferritin) was a better predictor of iron status and response to treatment with ESA in dialysis patients [6]. Our data revealed that only 11% of the variation in ferritin levels could be explained by the TSAT with 89% bearing no relationship. Although there was a statistically significant association between ferritin and TSAT on mixed regression analysis, a unit rise in TSAT was associated with a very small rise in ferritin which supports the correlation findings. This suggests that the high ferritin levels in our patients are only partly explained by iron levels. Our local iron management protocols have focussed on serum measures of TSAT and ferritin and are thus in accordance with other international anaemia management guidelines (Table 1). Yet we have shown that TSAT and ferritin are in fact likely to insufficiently direct optimal iron replacement, given the lower achieved haemoglobin among Indigenous clients in this cohort. Consequently, there is a clear need to explore other measures of iron status in addition to the ferritin and TSAT.

We recently reported that our patients, who have high serum ferritin concentrations, are also exposed to higher than usual levels of therapeutic iron doses [7], which is one consequence of a persisting evidence gap to guide management of iron deficiency in dialysis patients with high ferritin. In our patients, high serum ferritin concentrations, in combination with low TSAT, are observed prior to haemodialysis initiation. In contrast, data from the United States has shown that, with reductions in ESA use and increased iron supplementation, a rise in serum ferritin concentration among MHD patients is observed [10]. In the mixed regression analysis, we also showed no association between ferritin and ESA weekly dose, even though Indigenous patients required significantly higher doses of ESA.

We investigated the effect of inflammation, using CRP, as an alternate differential of high serum ferritin. Dialysis patients are known to have high vulnerability to bacterial infections [11], in our region, melioidosis bacteraemia [12], and staphylococcal bacteraemia [13]. In spite of this, our data showed that CRP was not associated with serum ferritin levels in maintenance haemodialysis patients. This suggests as yet unexplained causes of high serum ferritin, beyond infection in this population. This data was reported from a retrospective clinical dialysis database. Therefore, we did not have other more specific or novel measures of inflammation. We acknowledge that other markers of infectious and noninfectious inflammation may have been better surrogates of relationship between ferritin and inflammation. These may include more specific inflammatory markers such as interleukin-6 (IL-6) and tumour necrosis factor-a (TNF-a) [14].

Although the possibility of iron overload as a potential explanation for the lack of association between the high ferritin and CRP was considered, an analysis of the available data supported what we found in our other recent study that the administered iron did not explain the lack of association between the high ferritin and the CRP [7].

Although we performed sensitivity analyses which showed no difference in the association between ferritin and CRP and ferritin and infused iron dose in participants where the infused dose of iron was available compared to the findings observed in the combined cohort, the lack of association between ferritin and infused iron dose and ferritin and CRP may be explained by the low data for infused iron dose available in the dataset.

There is a significant potential risk of iatrogenic iron overload generated by the administration of iron in our dialysis patients with high ferritin where guidelines are lacking on the appropriate dosing in those with other evidence of iron deficiency. Although we have adjusted our local guidelines [7] in line with the findings in the Ferric Gluconate Is Highly Efficacious in Anaemic Haemodialysis Patients with High Serum Ferritin and Low Transferrin Saturation: Results of the Dialysis Patients' Response to IV Iron with Elevated Ferritin (DRIVE) studies [15, 16], a significant number of patients have the high ferritin levels above those used in these studies. Therefore, this needs further evaluation. A cross-sectional study assessing the best markers of iron stores in this population will be informative in helping to determine the best measures of the iron stores. However, the appropriate dosing of iron will need to be evaluated by a clinical trial comparing outcomes between the dosing of iron among different levels of high ferritin.

Dialysis adequacy as measured by URR was highly significantly associated with higher ferritin. This may reflect either better utilisation of iron in those patients who are dialysing well compared to those with poor dialysis or an indication that those who attend adequate dialysis receive iron treatment. This will need further assessment, including a prospective study as the question was beyond the scope of this study.

The lack of correlation with ferritin and TSAT suggests the need to examine the role of regulatory and functional markers of iron such as hepcidin [17–21] and other measures of iron stores, particularly soluble transferrin receptor which has been shown to be a better marker of iron status than ferritin in Indigenous children [22]. Other measures of iron stores such as low MCV and hypochromic, microcytic RBCs on blood film, percentage hypochromic red cells (PHRC), and reticulocyte haemoglobin content will need further exploration in prospective studies in this population. Although the PHRC produces comparable or somewhat better results than reticulocyte haemoglobin content, the blood sample needs to be analyzed on site, which makes its widespread adoption difficult in our setting [23].

Causes of very high serum ferritin concentrations (>10,000 *µ*g/l) include genetic causes, transfusional iron overload, juvenile idiopathic arthritis, lupus, and haemophagocytic lymphohistiocytosis. Iron overload may be another differential of high serum ferritin in this cohort, although our data did not support this among our patients, since the TSAT was not elevated. Direct assessments of iron status such as measuring liver iron levels could be considered [24]. The traditional invasive methods to determine body iron levels such as bone marrow biopsy [25] and liver biopsy are associated with increased mortality and morbidity [26]. However, the recent increase in the use of noninvasive but accurate methods such as the magnetic resonance imaging- (MRI-) based technique spin-density projection-assisted (SDPA) R2-MRI (FerriScan®) provides an opportunity to determine the relationship between the high ferritin and liver iron status to exclude iron overload [27–29].

Almost 20 years ago, Moirand et al. identified the relationship between unexplained high serum ferritin (median values > 500 *µ*g/l) and normal transferrin saturation [30]. They reported that the majority of participants with unexplained high serum ferritin had numerous clinical indicators of the metabolic syndrome [30]. This has also been supported more recently [31], with ferritin having strong association with insulin and c-peptide, which unfortunately were not routinely available in all our patients. It is reported that insulin increases externalisation of the transferrin receptor, thereby stimulating cellular iron uptake. Type 2 diabetes is a leading cause of end stage kidney disease in Indigenous Australians [32] and reflected in 66.6% of our participants. Hyperinsulinaemia and metabolic syndrome are also highly prevalent among the general population of Indigenous Australians in our region [33, 34]; hyperferritinaemia in the absence of acute infection may potentially reflect sustained hyperinsulinaemia among our patients. We therefore plan to investigate the association between high serum ferritin and markers of the metabolic syndrome in our MHD patients.

The study had several limitations including the retrospective design which is associated with potential bias due to reliance on data collected for a different aim to the study. However, there was enough information in the datasets to confidently assess the relationship between ferritin and the other variables to answer the question. Some important information on essential variables to this analysis was incomplete; for example, total dose of iron per annum had too few observations in the first few years of the data collection to be included in the analysis. This may have affected the levels of ferritin. However, this would not have affected the relationship between ferritin and TSAT as both are expected to rise with iron therapy. One of the limitations of the study as a retrospective analysis was that the data collection for iron infusion was not complete. In the earlier years covered by the study, the data entry was limited to whether iron infusion was given or not without specifying the dose. It was only in the last 12 months that the dose was included in the entry. Most of the analysis was performed on the whole sample and then by ethnicity. However, detail could not be provided on iron infusion and ferritin by ethnicity due to the limited available data.

A prospective study is also required to further comprehensively explore the potential causes of the high ferritin and the clinical outcomes that may be associated with the high ferritin in this patient group. Future studies to clarify these unanswered questions include a prospective study which combines assessing the association of ferritin with measures of regulatory and functional markers of iron status such as hepcidin, markers of inflammation mentioned above, and other surrogate methods of assessing iron status such as soluble transferrin receptor will provide a clear method of assessing iron status in our patients. Hepcidin is particularly increasingly recognised as a key player in iron metabolism and will need exploration in this population especially with respect to functional iron deficiency [17, 35]. An assessment of the relationship between ferritin and other clinical correlates such as hepatitis B infections, hospitalisations, cardiovascular disease, other infections, and conditions such as diabetes mellitus will also need further studies.

The implication of this study is that it raises questions on the veracity of using ferritin and transferrin saturation as the main determination of iron requirements and therapy in Indigenous patients on haemodialysis. The study clearly raises the need to further explore the use of other measures of iron stores. It also raises the need to conduct a clinical trial assessing the safety and efficacy of administering iron in patients with higher ferritin levels than in the DRIVE studies [15, 16].

## 5. Conclusion

We report a population of MHD clients with high ESA usage, hyperferritinaemia, and low-normal TSAT. Our data has revealed the limited utility of using only ferritin and TSAT to guide Renal Anaemia Management protocols in our region. We suggest exploring the utility of additional markers of iron status to guide our clinical practice. We have further shown the lack of correlation of ferritin with serum CRP, as a marker inflammation. There is clear need for further evaluation of the causes of this high ferritin and the possible associated detrimental clinical outcomes in these patients. Unlike populations without severe renal disease, we suspect that there may be an association between hyperferritinaemia and metabolic syndrome which could explain some of the variance in high serum ferritin concentrations in our patients. Should this be confirmed in future studies in our patients, then interventions targeting metabolic syndrome may also be incorporated into our local anaemia management guidelines.

## Figures and Tables

**Table 1:** Guidelines on target levels of markers of iron status in people with CKD.

Guidelines	Continent	Ferritin (*µ*g/l)	TSAT (%)
Canadian Renal Guidelines	Canada	100–500	>20
KDIGO	Worldwide	100–500	>20
ERA-EDTA	Europe	200–300	30–50
CARI	Australia & New Zealand	200–500	30–40
KDOQI	United States	200–500	30–50
UK Renal Association/NICE	United Kingdom	250–500	>20

CARI: Caring for Australasians with Renal Insufficiency, KDOQI: Kidney Disease Outcome Quality Initiative, UK Renal Association: United Kingdom Renal Association, NICE: National Institute for Health and Care Excellence, ERA-EDTA: European Renal Association-European Dialysis and Transplant Association, and KDIGO: Kidney Disease: Improving Global Outcomes [1, 7].

**Table 2:** Differences in variables by ethnicity.

Variable	Whole sample	Indigenous	Non-Indigenous	*p* value (by ethnicity)
Ferritin (*µ*g/l)	1022 (590–1491)	1069.5 (668–1522)	558.5 (199–1252)	<0.001
Transferrin saturation (%)	26 (19–37)	26 (19–37)	28 (20–38)	0.516
Haemoglobin (g/l)	107.6 (18.2)	106.6 (18.8)	113.2 (19.0)	<0.001
Potassium (mmol/l)	4.66 (0.86)	4.7 (0.9)	4.6 (0.8)	0.398
BMI (kg/m^2^)	26.8 (5.9)	26.3 (6.0)	26.4 (5.2)	0.259
CRP (mg/l)	13.9 (6.0–33.1)	14.7 (7–35)	5.9 (1.9–17.5)	<0.001
Corrected calcium (mmol/l)	2.27 (0.22)	2.26 (0.22)	2.32 (0.19)	<0.001
Albumin (g/l)	37.8 (5.5)	37.7 (5.5)	38.6 (5.4)	<0.001
Magnesium (mmol/l)	0.80 (0.14)	0.79 (0.13)	0.83 (0.16)	<0.001
Bicarbonate (mmol/l)	23.7 (4.6)	23.6 (4.7)	24.0 (4.1)	<0.001
Calcium phosphate product	3.67 (1.40)	3.66 (1.41)	3.75 (1.43)	0.163
Phosphate (mmol/l)	1.63 (0.65)	1.63 (0.65)	1.63 (0.63)	0.163
KT/V	1.61 (0.25)	1.61 (0.26)	1.61 (0.23)	0.171
URR (%)	74.6 (9.3)	74.8 (9.1)	73.2 (11.1)	0.010
ESA dose (IU per week)	11517 (8350)	11691 (8286)	9945 (8757)	<0.001

Data are median (IR) or mean (SD) as indicated.

**Table 3:** Bivariate association of ferritin and other factors using mixed methods.

Variable	Coefficient	*p* value	95% confidence interval
Male^*∗*^	0.05	0.590	−0.36–1.12
Non-Indigenous^*∗∗*^	−50.70	<0.001	−65.60–−37.95
Haemoglobin	0.00	0.069	0.00–0.00
Potassium	0.10	0.115	−0.03–0.23
Albumin	0.00	0.981	−0.02–0.02
Corrected calcium	−0.09	0.768	−0.69–0.51
Phosphate	0.17	0.085	−0.02–0.37
Calcium phosphate product	0.09	0.068	−0.01–0.18
Parathyroid hormone	0.001	0.800	−0.01–0.01
CRP	0.001	0.546	−0.001–0.003
Urea reduction ratio	0.06	<0.001	0.03–0.09
KTV	2.38	0.024	0.31–4.46
Age at the last review date	−0.06	<0.001	−0.09–−0.03
Body mass index	−0.08	0.009	−0.14–−0.02
Weekly darbepoetin dose	0.00	0.989	−0.01–0.01
Weekly epoetin alfa dose	0.00	0.891	0.00–0.00

^*∗*^Female is the baseline. ^*∗∗*^Indigenous is the baseline.

**Table 4:** The final model after stepwise sequential fitting of the model.

Variable	Change in square root of ferritin level^*∗*^	*p* value	95% confidence intervals
TSAT (%)	0.04	<0.001	0.04–0.05
Indigenous	(1.00)	—	—
Non-Indigenous	−13.47	<0.001	−29.16–3.80
URR	0.04	0.001	0.02–0.07

^*∗*^Change in the square root of ferritin per unit change in the independent variables.

**Table 5:** Results of two-level (multilevel) multinomial logistic regression: final model.

Predictor	Ferritin (*µ*g/l) categories^*∗*^		
	Category 2 ≥ 100 < 800	Category 3 ≥ 800 < 1200	Category 4 ≥ 1200
TSAT ≥ 20% and ≤30%	1.58	2.31	2.35
	<0.001	<0.001	<0.001
	1.39–1.77	2.12–2.51	2.15–2.54
TSAT > 30% and ≥50%	2.28	3.33	3.67
	<0.001	<0.001	<0.001
	1.98–2.57	3.03–3.63	3.37–3.96
TSAT > 50%	2.67	4.15	4.99
	<0.001	<0.001	<0.001
	2.02–3.32	3.50–4.80	4.34–5.64
Non-Indigenous ethnicity	−2.46	−3.43	−3.53
	<0.001	<0.001	<0.001
	−2.88–−2.04	−3.85–−3.00	−3.96–−3.11
Male gender	1.49	1.33	1.19
	<0.001	<0.001	<0.001
	1.13–1.86	0.96–1.70	0.82–1.56
CRP ≥ 10 mg/l	−0.04	−0.07	−0.09
	0.693	0.513	0.375
	−0.24–0.16	−0.28–0.14	−0.30–0.11

Data are coefficient, *p* value, and 95% confidence interval. Reference groups for categorical variables are TSAT < 20%, female gender, Indigenous ethnicity, and CRP < 10 mg. ^*∗*^Compared to base outcome of ferritin category 1 (serum ferritin < 100 *µ*g/l).

## Abbreviations

TSAT: Transferrin saturation
NT: Northern Territory
MHD: Maintenance haemodialysis
CRP: C-reactive protein
TIBC: Total iron binding capacity
ESA: Erythropoiesis stimulating agents
PHRC: Percentage hypochromic red cells
CARI: Caring for Australasians with Renal Insufficiency
KDOQI: Kidney Disease Outcome Quality Initiative
NICE: National Institute for Health and Clinical Excellence
ERA-EDTA: European Renal Association-European Dialysis and Transplant Association
KDIGO: Kidney Disease: Improving Global Outcomes
ESKD: End stage kidney disease
HREC: Human Research Ethics Committee
RAM: Renal Anaemia Management
BMI: Body mass index
URR: Urea reduction ratio
TNF: Tumour necrosis factor.
